# Head and neck cancer subtypes with biological and clinical relevance: Meta-analysis of gene-expression data

**DOI:** 10.18632/oncotarget.3301

**Published:** 2015-03-20

**Authors:** Loris De Cecco, Monica Nicolau, Marco Giannoccaro, Maria Grazia Daidone, Paolo Bossi, Laura Locati, Lisa Licitra, Silvana Canevari

**Affiliations:** ^1^ Functional Genomics and Bioinformatics, Dept. of Experimental Oncology and Molecular Medicine, Fondazione IRCCS Istituto Nazionale dei Tumori, Milan, Italy; ^2^ Department of Mathematics, Stanford University, Stanford, CA, USA; ^3^ Dept. of Experimental Oncology and Molecular Medicine, Fondazione IRCCS Istituto Nazionale dei Tumori, Milan, Italy; ^4^ Head and Neck Medical Oncology Unit, Fondazione IRCCS Istituto Nazionale dei Tumori, Milan, Italy

**Keywords:** tumor subtypes, gene expression, HNSCC, microarray, meta-analysis

## Abstract

Head and neck squamous cell carcinoma (HNSCC) is a disease with heterogeneous clinical behavior and response to therapies. Despite the introduction of multimodality treatment, 40–50% of patients with advanced disease recur. Therefore, there is an urgent need to improve the classification beyond the current parameters in clinical use to better stratify patients and the therapeutic approaches. Following a meta-analysis approach we built a large training set to whom we applied a Disease-Specific Genomic Analysis (DSGA) to identify the disease component embedded into the tumor data. Eleven independent microarray datasets were used as validation sets.

Six different HNSCC subtypes that summarize the aberrant alterations occurring during tumor progression were identified. Based on their main biological characteristics and de-regulated signaling pathways, the subtypes were designed as immunoreactive, inflammatory, human papilloma virus (HPV)-like, classical, hypoxia associated, and mesenchymal. Our findings highlighted a more aggressive behavior for mesenchymal and hypoxia-associated subtypes. The Genomics Drug Sensitivity Project was used to identify potential associations with drug sensitivity and significant differences were observed among the six subtypes.

To conclude, we report a robust molecularly defined subtype classification in HNSCC that can improve patient selection and pave the way to the development of appropriate therapeutic strategies.

## INTRODUCTION

Head and neck squamous cell carcinoma (HNSCC) is a heterogeneous set of distinct malignancies. Recognized prognostic factors rely on clinical and biological features, consisting mainly of stage, site of disease, performance status, comorbidities, smoking history and human papilloma virus (HPV) status [1]. However, patients clustered by these parameters still differ in their clinical behavior and therapy response [2, 3].

Advancements in genomic technologies have allowed the identification of different genomic and epigenomic alterations formed during transformation and tumor progression. Eventually, the improvement in our understanding of complex heterogeneity of human tumors is expected to lead to more individualized therapies and targeted drug design. An efficient way to decipher cancer heterogeneity is to identify subtypes driven by molecular patterns and develop a classifier to predict the subtype membership of a new sample.

Microarray technology has allowed researches to exploit the whole transcriptome landscape to define new molecular cancer subtypes, undetected by the traditional histopathological parameters. According to these advancements, numerous studies have dissected gene expression profiles to identify clusters of patients with common molecular patterns in different tumor types. This approach started in breast cancer by the pioneering work at Stanford University [4] and after more than a decade it is clear that at least five molecular subtypes showing clinical relevance are present. In 2001, Sorlie and colleagues defined subtype signatures in intrinsic genes identified by analysis of before-and-after chemotherapy treatment and obtained breast cancer molecular subtypes, which were later validated in independent cohorts [5]. Subsequently, the signature was refined [6] and its last version, PAM50, added prognostic and predictive value to the traditional pathologic, histological, and biological parameters [7]. After this first approach in breast cancer, the interest in subtype discovery has continuously grown and, at present, a number of different malignancies including lung [8], colorectal [9], brain [10], gastric [11], and pancreatic [12] cancer has been investigated. A typical workflow involves some key steps such as the identification of subtypes through appropriate bioinformatics methods, the development of a classifier, and validation in external datasets. Since it is unknown the number and the relative occurrence of subtypes, the size of initial discovery cohort has a paramount importance to be confident in identifying even rare subtypes. As a consequence, the most recent work includes a training set ranging from 500 to 1000 cases.

The integration of multiple datasets exploiting a meta-analysis approach has been reported to offer invaluable advantages, improving the reliability of results, especially for HNSCC, for which few microarray datasets, with frequently a limited number of cases, are publicly available. In addition, through meta-analysis it is possible to reach an adequate sample size allowing detection of rare subtypes unlikely to be seen in small patient series. For instance, the merger of gene-expression datasets in ovarian cancer [13] in a meta-analysis of approximately 1500 cases derived from 16 studies enabled the identification of five reliable subtypes with unique outcomes.

In the last decade, there has been a continuous development in methods for data analysis leading to innovative bioinformatics approaches for data decomposition. Among them, Disease-Specific Genomic Analysis (DSGA) [14] allows defining a Healthy State Model (HSM) from the expression data of normal tissues and based on that, the disease component is computed as the residuals between the tumor and normal components.

Here, we report a genomic approach to dissect the heterogeneity of HNSCC. We established a large-scale meta-analysis approach followed by data decomposition through DSGA to identify HNSCC unique molecular subtypes. Our findings were validated in independent datasets and our classification reveals the presence of six subgroups with distinct biology and clinical outcome.

## RESULTS

**Figure 1:**
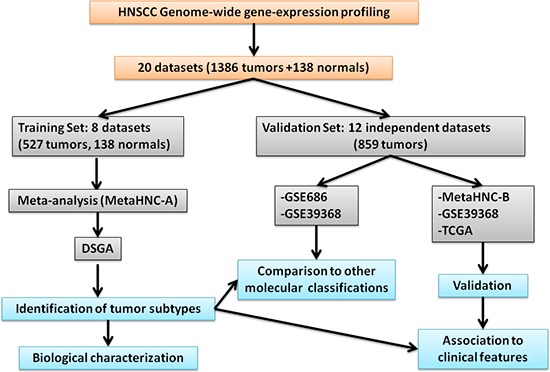
Study outline

Figure 1 shows the outline of our study. A systematic search in the PubMed database (http://www.ncbi.nlm.nih.gov/pubmed) (January 2000 to December 2013) for studies on head and neck cancer reporting gene expression data was performed. As selection criteria, we impose that the studies include: (i) squamous cell carcinoma primary lesions; (ii) tumor location including oral cavity, pharynx, and larynx (salivary glands, thyroid, and eyes were excluded); (iii) gene expression profiling of at least 15 samples. In this way we were able to select 30 studies. Subsequently, among them we focused our attention on those that reported: (i) MIAME [15] compliant datasets including raw and/or processed microarray data deposited on publicly accessible repositories and full gene annotation (Gene Bank accession or EntrezID); (ii) clinical data associated to microarray data. Based on these selection criteria, 20 datasets (Table S1) were retrieved listing 1386 tumor samples and 138 normal tissue samples. Eight datasets, profiled on Affymetrix HG-133_plus_2 arrays were used to generate a meta-analysis training set and the remaining 12 datasets served as validation sets.

### Unsupervised analysis revealed six subtypes in HNSCC

To analyze the molecular heterogeneity of HNSCC, we established a large meta-analysis of publicly available gene-expression datasets. The expression data of 527 tumor cases along with 138 normal cases belonging to eight different datasets were integrated into a single unified dataset, hereafter named MetaHNC-A.

First, we applied a data structure decomposition approach through DSGA (Figure S1). The expression microarray data of normal tissues allows definition of the HSM, which reflects the healthy tissue. Based on HSM, each tumor tissue is decomposed as the sum of two components: (i) the normal component, its linear model fit to the HSM; (ii) the disease component, vector of residuals, assessing the extent to which each tumor deviates from the normal state. The disease component was used for the identification of the molecular subtypes.

**Figure 2:**
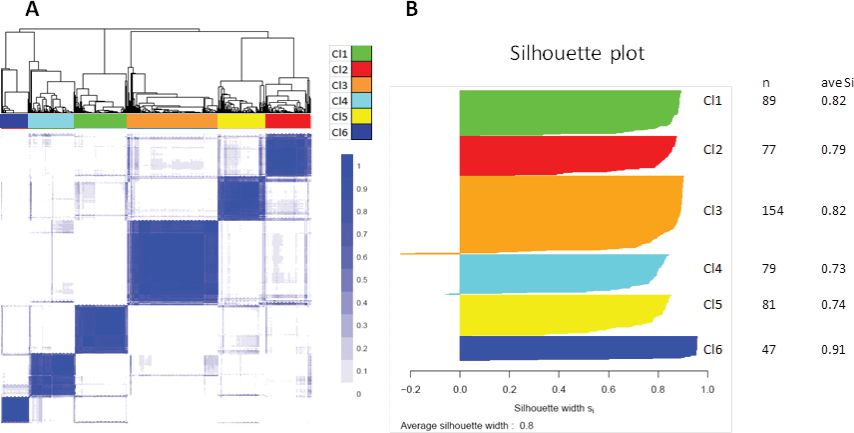
Molecular classification in HNSCC Results are produced by ConsensusClusterPlus for 527 cases on 4950 most variable genes. **A.** Consensus matrix heatmap imposing six subtypes on the dataset: Cl1 (*n* = 89; 17%); Cl2 (*n* = 77; 15%); Cl3 (*n* = 154; 29%); Cl4 (*n* = 79; 15%); Cl5 (*n* = 81; 15%); Cl6 (*n* = 47; 9%). The consensus values range from 0 (white, samples that never cluster together) to 1 (blue, samples showing high clustering affinity). **B.** Silhouette plot analysis. Since the actual number of subtypes in HNSCC is not known, we should take into account that the number of subtypes may be greater than six with some subtypes not sufficiently represented in our dataset. To ascertain whether some samples are forced to belong to a certain cluster, silhouette plot analysis was carried out. The widths indicate a strong similarity of the samples within their subgroup compared with the samples belonging to other subgroups.

Consensus unsupervised clustering was applied to the disease component, taking into account the most variant genes of the MetaHNC-A training set, and revealed six clusters of samples (Figure 2A). The consensus heatmap provided evidence that the six clusters appeared well-defined. In our analysis, although a different number of clusters (*k*) produced reasonable stability, an increase in cluster stability was observed for *k* ranging from 2 to 6 and the CDF becomes stable with balanced partitions. When *k* was >7, only marginal gains were observed (Figure S2).

To assess the accuracy of our classification, a Silhouette plot analysis was carried out. As shown in Figure 2B, only a minimal number of tumors in the Cl3 and Cl4 subgroups were not assigned to the cluster, indicating the robustness of the classification. This result was also supported by cluster significance analysis through SigClust (Table S2) and by evaluation of the sample size adequacy that reaches enough power for the detection of the six subtypes (Figure S3).

### Functional annotation of HNSCC subtypes

**Figure 3:**
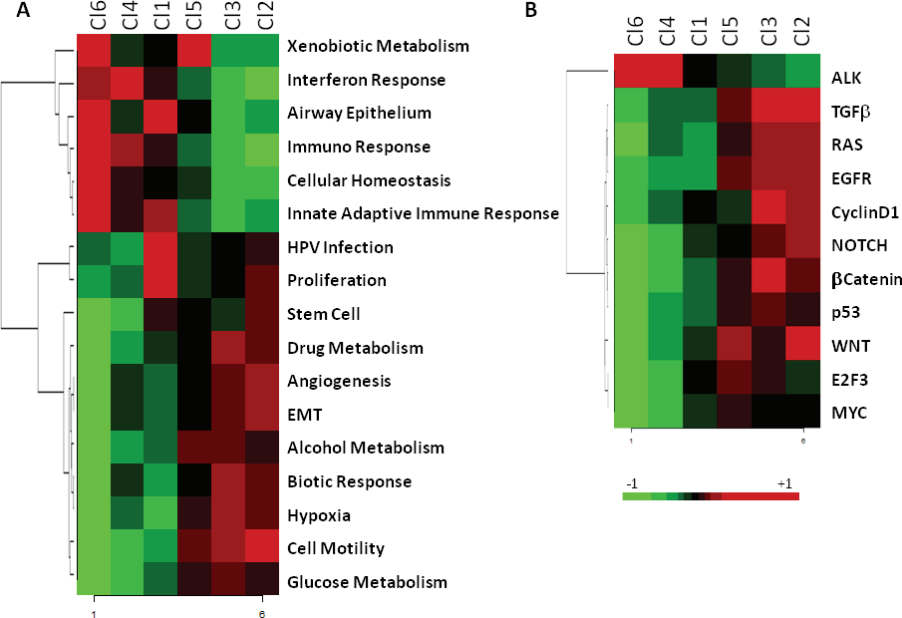
Heatmap of pathways enriched in the six subtypes The molecular pathways and onco-signatures enriched in each subtype as investigated through GSEA. **A.** The relative enrichment of 17 gene-ontology pathways related to biological processes. **B.** The relative enrichment of 11 onco-signatures.

The biological pathways related to each subtype were investigated using gene set enrichment analysis (GSEA). The results are displayed in Figure 3 and summarized in Table 1. In the C11 subgroup the up regulated genes were related to HPV infection and cell proliferation (Figure 3A). The Cl2 subgroup showed marked enrichment of a number of pathways including epithelial mesenchymal transition (EMT), cell motility, angiogenesis, and in the genes belonging to WNT and Notch onco-signatures (Figure 3A and 3B). The Cl3 subgroup was showed enhancement in hypoxia, drug metabolism pathways, and the genes belonging to beta-catenin pathway (Figure 3A and 3B). Furthermore, both the Cl2 and Cl3 subgroups, compared with the other four, showed an up-regulation of genes belonging to pathways involving tumor growth factor β (TGFβ), rat sarcoma (RAS), epidermal growth factor receptor (EGFR), and Cyclin D1 (Figure 3B). The Cl4 subgroup showed enrichment in the interferon response pathway (Figure 3A), immune response (Figure 3A), and genes belonging to ALK onco-signature (Figure 3B). The Cl5 subgroup was mainly characterized by increased expression of genes related to the smoking related pathway (xenobiotic metabolism) (Figure 3A). The Cl6 subgroup also expressed up-regulation of all of the immune system related pathways and was specifically enriched in cellular homeostasis and cellular markers specific of air way epithelium (Figure 3A).

**Table 1:** Summary of the main characteristics of the identified HNSCC subtypes

Association to:	HNSCC subtypes ordered according to progression of disease					
	Cluster 6	Cluster 4	Cluster 1	Cluster 5	Cluster 3	Cluster 2
Functional pathways	IFN response Immune response Airway epithelium Cellular Homeostasis Xenobiotic met.	IFN response Immune response	HPV infection Cell proliferation Airway epithelium	Cell motility Xenobiotic met.	Cell motility Hypoxia Drug metabolismBiotic response	Cell motility EMT Angiogenesis
Onco-signatures	ALK	ALK	None	Multiple:WNT E2F3 TGF beta	Multiple:TGF beta EGFR Ras Cyclin D1	Multiple:WNT TGF beta EGFR Ras NOTCH
Previously reported subtypes	AT	BA, G1	AT, G3	CL, G4	BA, G1	MS, G2
Clinic-pathological parameters			Oropharynx cases	Smoking		
Outcome			Best RFS Best OS		Worst RFS Worst OS	Worst RFS Worst OS
Previously reported classifiers			Best outcome		Worst outcome	Worst outcome
Final designation	Immunoreactive	Defense response	HPV-like	Classical	Hypoxia	Mesenchymal

Based on the biological features, we defined the six subtypes as: HPV-like (Cl1), Mesenchymal (Cl2), Hypoxia-associated (Cl3), Defense response (Cl4), Classical (Cl5), and Immunoreactive (Cl6).

### Comparison to previous molecular classifications

**Figure 4:**
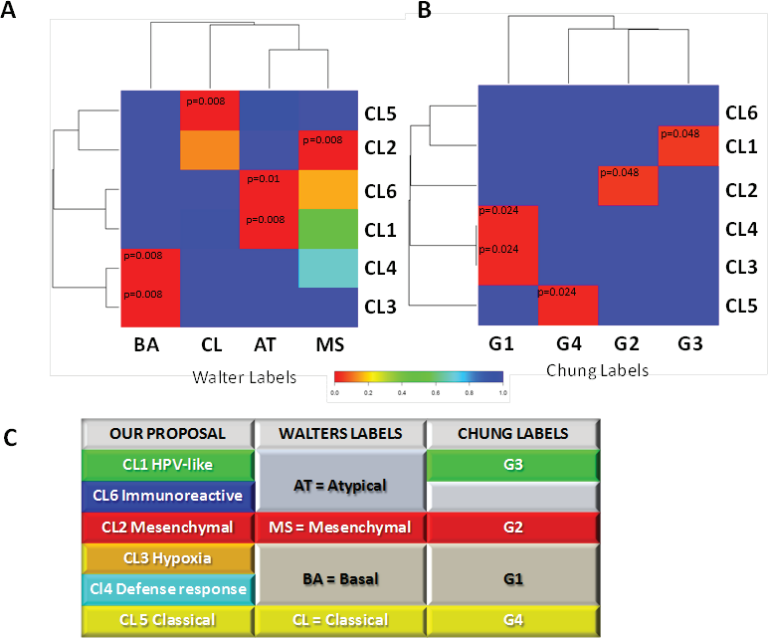
Comparison of genome-wide molecular pattern between our and previously reported subtype classification The analysis was performed using Subclass Mapping. **A.** MetaHNC-A is compared with the molecular subtypes defined by Walter *et al*. ((48); GSE39368). **B.** MetaHNC-A is compared to the subtypes reported by Chung *et al*. ((47); GSE686). Red color indicates high confidence for correspondence (*p* < 0.05); blue color indicates lack of correspondence. BA, basal; MS, mesenchymal; AT, atypical; CL, classical subtypes in the study by Walter *et al*. G1, G2, G3, G4 refer to the four subtypes identified in the study by Chung *et al*. **C.** Table summarizing the correspondence between our subtyping classification and those previously published for HNSCC by Chung *et al.* (47) and Walter *et al.* (48).

We investigated whether and to what extent the molecular classification described in the present study corresponded to those reported by the two previous studies addressing this issue, Chung *et al*. [16] and Walter *et al*. [17]. By Subclass Mapping, we assessed the overall concordance comparing: (i) the classification outlined above to that of Walter *et al*. (Figure 4A); (ii) he classification outlined above to that of Chung *et al*. (Figure 4B). The subtyping scheme from the previous studies did not show a one-to-one match with classification outlined above (Figure 4C), providing evidence that our meta-analysis is able to add a finer distinction not achievable with fewer samples (*n* = 60 for GSE686 and *n* = 138 for GSE39368). Whilst the Mesenchymal and Classical classifications proposed by Walter *et al*. and the G2 and G4 subtypes proposed by Chung *et al*. correspond to our Cl2-Mesenchymal and Cl5-Classical, the Basal and G1 subtypes proposed by Walter *et al.* and Chung *et al*. respectively showed molecular patterns split between our Cl3-Hypoxia associated and Cl4-Defense response subtypes. Furthermore, the atypical subtype proposed by Walter *et al*. is split between our Cl1-HPV-like and Cl6-immunoreactive subtypes, whereas the G3 subtype proposed by Chung *et al*. corresponds to the Cl1-HPV-like cluster.

### Progression analysis of disease

**Figure 5:**
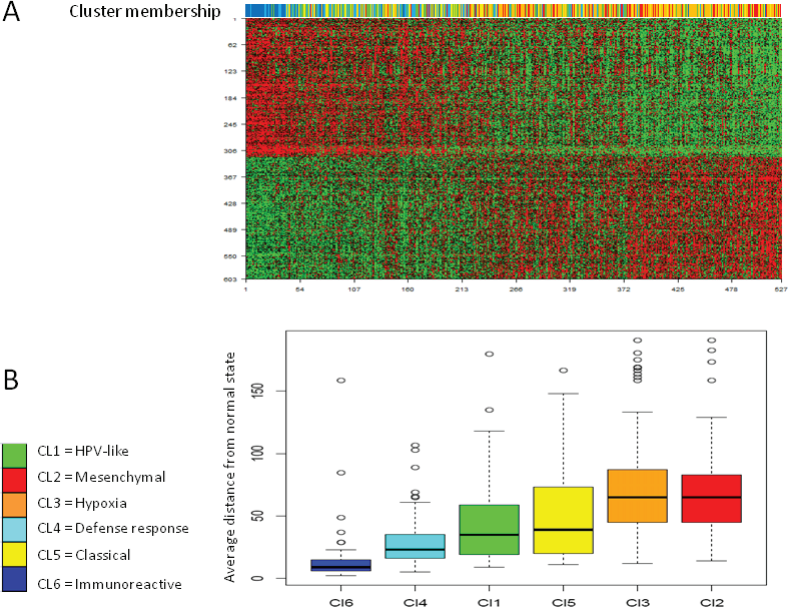
Progression analysis of disease The average distance of each tumor from the normal state has been assessed. **A.** 603 genes were identified associated to PAD. The upper bar illustrates to which subtype belongs each tumor sample. **B.** The box plots show the distance from normal state of each tumor was in relation to the six subtypes. *Y*-axis represents the distance from normal state computed as average bin-membership by PAD and depicted in Figure S4.

We applied Mapper [18] a tool able to capture topological and geometric shapes in complex multidimensional data and included in PAD software, to the DSGA-transformed data matrix computed on the 527 cases in our meta-analysis. Figure S4 shows the output of PAD analysis. HNSCC tumors can be associated through a linear progression starting from tumors displaying features close to the normal state (blue bins) and ending with tumors with large deviation from the normal state (red bins), suggesting an increase in alterations accumulated during different stages of tumor progression. Through PAD analysis, 603 genes were found to significantly correlate to tumor progression (Figure 5A). The genes negatively correlated to PAD (i.e. up-regulated in tumors close to the normal state) were enriched in chemokines and cytokine indicating a huge communication among tumor cells and stroma. As the disease progresses, tumors present genes positively correlated to PAD and encode proteins related to tumor plasticity, invasion, and metastasis. Functional analysis of signaling pathways and network connections were performed by IPA. The top molecular functions (imposing a score >30) are illustrated in Figure S5.

Since each tumor can occur in different bins within PAD analysis, we established an average position for all samples and compared the results to the six subtype classification. The subtypes described here summarized the tumor progression established by PAD with the Cl6 subtype displaying a molecular pattern close to the normal state, while the Cl2 and Cl3 subtypes were the most distant (Figure 5B).

### Validation of the subtypes across two HNSCC datasets

**Figure 6:**
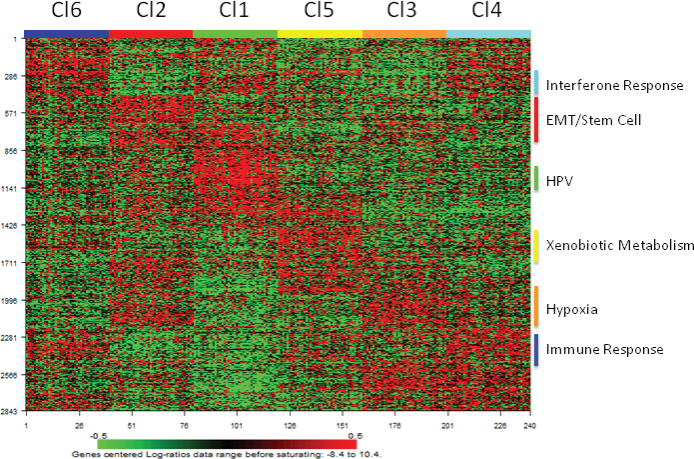
Distribution of the PAM classifier genes in the HNSCC subtypes identified in the training dataset Heatmap of the expression values of the 2843 classifier genes.

Eleven independent datasets were retrieved from public domains (GEO and TCGA). Two datasets, GSE39368 and TCGA, were profiled on Agilent and Illumina RNAseq platforms, respectively. The remaining nine datasets comprising a total of 358 samples were profiled on different types of chips belonging to the Affymetrix platform and were computationally integrated through a meta-analysis approach to build a unique independent validation set, hereafter named MetaHNC-B (Figure 1). The subtype membership on these datasets was predicted using PAM. First, we developed a prediction algorithm based on PAM using 40 ‘core samples’ for each subtype as established by Silhouette analysis. A total of 2843 genes entered into the classifier, yielding a cross-validation mis-classification rate of 5%. Figure 6 shows the heatmap of the classifier genes on MetaHNC-A, providing evidence that each subtype has its own distinct expression pattern. The list of genes, shrunken centroid values for each subtype and the algorithm to classify a new sample are reported in Table S3.

This classifier was applied to GSE39368, TCGA and MetaHNC-B (Figure 1) datasets and the validation sets clearly recapitulate the six cluster classification (Figure S6A, S6C, and S6E). Through Subclass Mapping we confirmed a good molecular correspondence (*p* < 0.05) of our classification in the external datasets (Figure S6B, S6D, and S6F).

### Association with clinicopathological parameters

The association between the six subtypes and tumor characteristics was investigated in the GSE39368 and TCGA validation datasets that were considered as reporting an appropriate number of cases and representative of the population in clinical practice. We assessed the proportion of cases within each subtype in relation to: (i) gender; (ii) alcohol consumption; (iii) smoking; (iv) pathologic stage; (v) pathologic T; (vi) pathologic N; (vii) tumor site (Figure S7). In both datasets, we found an association for tumor site and smoking history. The Cl5 subtype showed a significant presence of patients with heavy smoking history compared to the other subtypes, consistent with the GSEA functional analysis; the C11 subtype contained a greater number of oropharynx cases (~70%) (Figure S7).

A recursive partitioning approach was applied to ascertain to what extent the six subtypes can be predicted by exploiting exclusively the data of known clinical and pathological parameters. Gender, age, smoking history, pathologic stage, and site of primary tumor were included to build a classification tree on TCGA and GSE39368 datasets. The terminal nodes of the tree fail to identify unequivocally the six subtypes (Figure S8). Nevertheless, an increased occurrence in oropharynx tumors is associated to the Cl1 subtype reflecting the high presence of HPV positive cases. Altogether, this provides evidence that our gene-expression based classification adds a new layer of information not captured by the conventional clinical/pathological parameters.

### Prognostic value of the six-subtype classification

**Figure 7:**
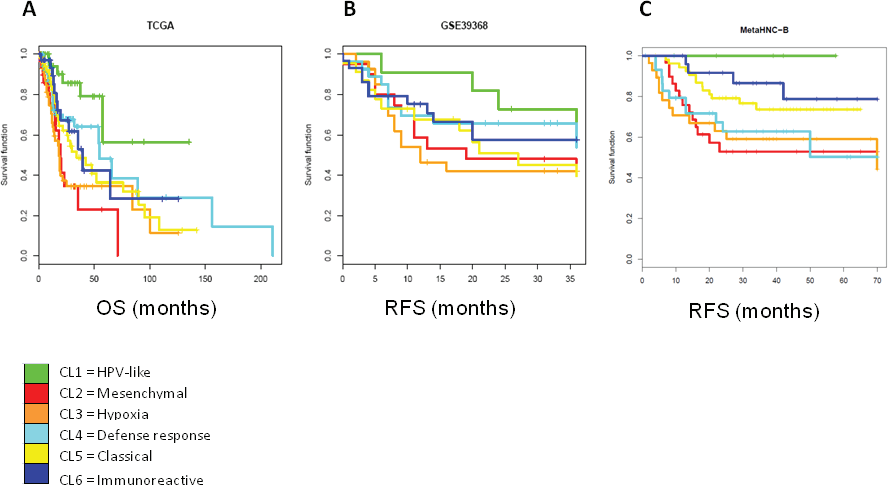
Survival analysis by Kaplan-Meier for each subtype The cases entering into the six subtypes identified on both validation datasets were used for the Kaplan-Meier analysis. **A.** TCGA dataset: log rank *p* = 0.0006; **B.** GSE39368 dataset: log rank *p* = 0.576; **C.** MetaHNC-B dataset: log rank *p* = 0.0312. OS, overall survival; RFS, relapse free survival.

The clinical relevance of our classification was investigated and associated to the outcome in the three external validation datasets. We found that the six subtype stratification provides useful prognostic information. As a matter of fact, the prognostic value of the six subtype classifications was significant in the TCGA dataset, with better outcome for patients belonging to Cl1 subtype and worse for Cl2 and Cl3 subtypes (Figure 7A) (*p* = 0.0006). On GSE39368, although the prognosis of each of the six subtypes differed, it failed to reach a significant value. Nonetheless, we confirmed that patients belonging to the Cl1 subtype showed a better outcome compared with those belonging to the Cl2 or Cl3 subtypes (Figure 7B) (2 years RFS proportion of 72.7% for the Cl1 subtype compared with 48% and 42.1% for the Cl2 and Cl3 subtypes, respectively). A significant correlation (*p* = 0.0312) was observed in the MetaHNC-B dataset for a positive prognosis of the C11 subtype and a negative prognosis for the C12 and C13 subtypes (Figure 7C).

In recent years several gene-expression signatures have been reported as promising prognostic models in HNSCC. The relationship between the six subtypes and four classifiers (radiosensitivity index (RSI) [19]; 15-gene hypoxia classifier [20]; 13-gene signature for HPV-negative OSCC [21]; 172-gene model [22]) demonstrated a significant relationship of our stratification to these molecular signatures (Figure S9). Specifically, cases belonging to the Cl2 and Cl3 subtypes show the highest predicted risk, whereas the Cl1 cases show the better clinical outcome.

### Drug sensitivity of the six subtypes

**Figure 8:**
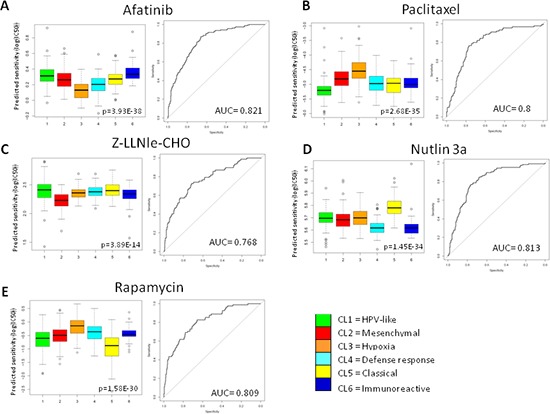
Prediction drug sensitivity in HNSCC subtypes Drug sensitivity was predicted for each case entering the MetaHNC-A dataset. Five therapeutic agents were investigated: **A.** Afatinib; **B.** Paclitaxel; **C.** Z-LLNle-CHO; **D.** Nutlin 3a; **E.** Rapamycin. Box-plots depict the predicted drug sensitivity in the six subtypes and the ROC curves estimate prediction accuracy of the more sensitive subtype against the others. *p* = Kruskal-Wallis test; AUC, area under the curve.

The Genomics Drug Sensitivity Project [23] includes gene-expression profiling data of hundreds of cancer cell lines along with sensitivity data to 130 drugs. We tested the possibility that each subtype might have specific drug sensitivity, applying a phenotype prediction machine learning tool matching cell line chemotherapeutic response to baseline tumor gene expression [24]. As proof of concept, we restricted our analysis to 46 cell lines defined as ‘upper aerodigestive’ and to a list of drugs in clinical use or under preclinical investigation in HNSCC including Paclitaxel, Rapamycin, Afatinib, Nutlin3a, and Z-LLNle-CHO. Our findings demonstrated a statistically significant difference in drug sensitivity for patients belonging to different subtypes. As example, EGFR inhibitors have received great interest in HNSCC but at present the response rate is less than 15% [25]. Our results reported in Figure 8 strongly suggest that Cl3 subtype shows greater sensitivity to Afatinib compared to the others and those patients could benefit from the treatment (Figure 8A). In addition, Figure 8 reports drug sensitivity for Paclitaxel, Z-LLNle-CHO, Nutlin3a, and Rapamycin. On this basis it may be predicted the drug potentially more effective for each subtype: Paclitaxel for Cl1 subtype (Figure 8B); Z-LLNle-CHO for Cl2 subtype (Figure 8C); Nutlin3a for Cl4 and Cl6 subtypes (Figure 8D); and Rapamycin for Cl5 subtype (Figure 8E).

## DISCUSSION

The workflow for cancer subtype identification involves some key steps such as application of appropriate bioinformatics methods, development of a classifier, and validation on external datasets. By applying this workflow to a meta-analysis of gene expression microarray datasets followed by data decomposition and throughout validation, we were able to define a robust genomic classification of HNSCC that could potentially identify targetable biological pathways, relevant clinical parameters, and eventually relate to clinical outcome.

Large datasets are required in order to characterize tumor subtypes especially when present at low frequency, but in malignancies like HNSCC accounting for about 5% of adult tumors, only a limited number of MIAME complaint datasets are publicly available. In this context, a meta-analysis approach combining multiple datasets together might help to overcome the issue, improving the reliability of results. Taking advantage of the availability of expression profiles of the normal counterpart we applied a data decomposition approach by DSGA, a proven method to improve the understanding of the biology underlying a pathologic process, to our training set. Our training set, MetaHNC-A, built by a meta-analysis approach, enabled the assessment of the disease state computed as the deviation of each tumor sample from the normal phenotype allowing the identification of six subtypes with sample size ensuring that at least 78% of genes achieve 90% power. Furthermore, DSGA followed by Mapper analysis (PAD analysis) revealed the topological connections among samples as a function of the gene-expression data and it was found that the six molecular subtypes summarize the continuous progression from samples close (in the Cl6 and Cl4 subtypes) to samples distant (in the Cl3 and Cl2 subtypes) to the normal state and that 603 genes showed a significant correlation to PAD analysis. Finally, the good performance of our classification in the validation sets provided evidence that our findings are not impaired by technical biases, laboratory or sample collection procedures.

The importance in defining molecular subtypes in HNSCC lies in their ability to provide new insights not captured by the known clinical/pathological parameters and to identify potentially targetable biological pathways. Thus, following the disease progression identified here, the main biological and clinical characteristics of each subtype (Table 1) and their predicted sensitivity to selected drugs under clinical evaluation in HNSCC (Figure 8) are hereafter commented in the context of literature data and of their potential therapeutic implications.

Cluster 6, the closest to the normal state according to the PAD analysis, expressed similarity with the airway epithelium and is the only one that maintains an active cellular homeostasis. This subtype, at variance to all the other ones, is characterized by activation of several immune-related pathways and accordingly we gave it ‘immunoreactive’ as its final designation. Interestingly, only the ALK onco-signature was activated compared with the multiple onco-signature activation present in the subtypes more distant to the normal state. Despite the apparently limited alterations of signaling pathways, the outcome of patients belonging to this cluster was not as good as could be expected. Since the Cl6 subtype is the smaller cluster and the only one not reaching 90% power for all genes, the analysis of a larger case series is needed to confirm the molecular features of this subtype.

Cluster 4, more distant from normal state compared with Cl6, as highlighted not only by DSGA but also by the loss of normal traits such as the pathways of airway epithelium and cellular homeostasis, presented the highest activation of the interferon response and high levels of immune response and ALK onco-signature. On this basis we assigned ‘defense response’ as its final designation. The absence of association with known clinical parameters and the different outcome of Cl4 patients observed in the three datasets analyzed seem to indicate that this new biologically distinct subtype required further molecular characterization.

However, if the identified Cl6 and Cl4 molecular portraits are confirmed, new immunotherapeutic modalities and/or ALK targeted therapy [26] that have never been analyzed in HNSCC, could be offered to patients with high immunoreactivity (Cl6) or defense response status and ALK activation (Cl4). Furthermore the higher sensitivity to Nutlin 3a, in agreement with the deactivated p53 onco-signature (Figure 3), suggests considering the patients in these two subtypes as eligible to combined treatment with Nutlin 3a as activator of p53-mediated apoptosis.

Cluster 1 was the only one with activation of HPV and proliferation signatures and no onco-signatures. Furthermore, cluster 1 maintained the immune systems active status and expressed similarity with the airway epithelium. When a classification tree based on recursive partitioning method was applied to the five available clinical parameters (gender, age, smoking history, pathologic stage, and site of primary tumor), Cl1 showed a significant enrichment in oropharyngeal cases. Even if the molecular identification of HPV infection in the analyzed MetaHNC-A dataset was not available, altogether the observed molecular portrait seemed to reflect a HPV positive status therefore resulting in Cl1 being classified as ‘HPV-like’. In agreement with data related to HPV cases [27], Cl1 showed the best outcome and was associated with signatures indicating a good prognosis: high radio-sensitivity [19], high similarity to the ‘less’ hypoxic group [20], low 13-gene OSCC [21] and low 172-gene model risk score [22]. Interestingly, even if we tested only one conventional drug (Paclitaxel) to predict sensitivity, the Cl1 subtype had the highest sensitivity (Figure 8), consistent with the specific activation of the proliferation pathway in this subtype.

Cluster 5 was identified by our approach characterized by xenobiotic response associated to smoking injury and moderate activation of cell motility, WNT, and E2F3 onco-signatures. Compared with the other subtypes, cluster 5 showed a significant presence of patients with the heaviest smoking history. These data, together with the high concordance of this subtype to the previous classifications lead to us to classify cluster 5 as ‘Classical’ for its final designation. The medium level of modification in WNT and E2F3 onco-signatures and the highest predicted sensitivity to Rapamycin might open the way to investigating at pre-clinical level the potential therapeutic activity of new oncogene-inhibitors and suggest the use of inhibitors of the mTOR pathway, whose alteration has been associated to smoke injury [28], in patients belonging to Cl5 subtype.

Cluster 3 was characterized by specific activation of drug metabolism and hypoxia pathways and according to its tumor progression association [29] shared with Cl2 cell motility; furthermore multiple onco-signatures were activated as a result. According to the peculiar activation of the hypoxia signature we gave it ‘Hypoxia’ as the final designation.

Cluster 2, the most distant form the normal state on the basis of PAD analysis, showed the highest cell motility expression accompanied by activation of EMT, angiogenesis and stem cell signatures. These data, together with the high concordance of this subtype to the previous classifications, brought us to ‘Mesenchymal’ as its final designation.

Both Cl3 and Cl2 showed a poor outcome in TCGA and MetaHNC-B datasets and, when analyzed with signature/classifiers reflecting poor prognosis, both resulted in high radio-resistance [19], showed similarity to the ‘more’ hypoxic groups [20], presented a high 13-gene OSCC score [21], and a high 172-gene model risk [22] score. The continuum in the disease progression identified by PAD analysis was demonstrated by the multiple alterations observed in Cl3 and Cl2, including seven out of 12 different members of the melanoma antigen gene family A (MAGEA), previously reported to induce growth by inhibition of cell cycle arrest and apoptosis [30]. Notably, similarly to MAGEAs, EGFR, an important therapeutic target in HNSCC, is highly expressed in tumors distant to the normal phenotype, defining the groups of patients that could benefit from EGFR-inhibitors [31]. The overall analysis for C2 and Cl3 clearly identified specific molecular portraits and predicted drug sensitivity that could in future be exploited for evaluating the impact of specific targeted therapies. In particular for the Cl3 subtype the presence of an EGFR activated pathway, in line with the predicted high sensitivity to Afatinib and the identification of altered hypoxia pathway, suggest the use of EGFR-targeted therapies combined with anti-oxidant agents and/or additional strategies exploiting hypoxia, as already suggested some years ago [29]; regarding the Cl2 subtype, the altered angiogenesis pathway and the activation of NOTCH pathway, in line with the predicted high sensitivity to Z-LLNle-CHO, supports the clinical evaluation of angiogenesis-targeted therapies.

In conclusion, the application of DSGA for the first time to pathology other than breast cancer enabled the description of a robust transcriptome-based subtype classification of HNSCC that improved the current clinicopathological and genome-wide stratifications. Notably, our meta-analysis study was able to disclose an improved molecular stratification in not previously seen subgroups characterized by distinct features.

Our comprehensive gene-expression classification of HNSCC offers some groundwork to the scientific community to improve the knowledge in the molecular pathways de-regulated in this disease. Hopefully, upon validation in prospective cohorts from clinical trials, the new, further refined, classification may result in personalized therapies for homogenous groups of patients.

## METHODS

### Data processing

Eight datasets profiled on the same array platform were selected to build a uniform training set through a meta-analysis approach (MetaHNC-A). Briefly, raw microarray data were retrieved from NCBI Gene Expression Omnibus (GEO) database [21, 32–36], ArrayExpress (The EMBL-European Bioinformatics Institute, UK) [37], and MIAME-Vice [38] repositories. See Table S1 for details regarding the datasets including the accession numbers.

First, signal intensity was normalized within each individual dataset using a Robust Multi-Array Average (RMA) tool. To reduce the likelihood of systemic non-biological technical experimental biases causing batch effects, the normalizing algorithm ComBat was applied [39]. The resulting dataset containing 665 samples was used for the analysis. Redundancy of probes mapping the same EntrezID was removed by selecting the probe having highest variance across samples using collapse Row R function [40]. Finally, a decomposition method intended to precede any further analysis was applied to the dataset. We used DSGA [14], unravelling the disease features embedded to the expression data of tumor samples (Figure S1); this method defines HSM from the expression data of normal tissues through FLAT construction and Principal Component Analysis [14]. This method permits each tumor tissue to define a model for its own normal component allowing modelling of the intrinsic diversity of normal tissue. The data matrix corresponding to the disease component was filtered in order to exclude the genes whose variation is below the 75^th^ percentile of the median variance of all genes, yielding 4950 unique EntrezIDs.

For validation purposes, 12 datasets profiled in different platforms were used (Table S1). GSE39368 and TCGA, including 138 and 303 samples respectively, were used to provide a molecular confirmation of our subtype classification along with an evaluation of the associations to clinical parameters. TCGA's level 3 files were downloaded along with the clinical annotations in June 2013 from the TCGA website (http://cancergenome.nih.gov/) and used for the analysis. For GSE39368 and GSE686, the processed data matrices available on GEO were retrieved and missing values present on GSE686 were imputed through the ImputeMissingValuesKNN module present in GenePattern software (Broad Institute, MIT, USA). The remaining nine datasets (GSE2379, GSE2837, GSE3292, GSE3524, GSE6631, GSE9349, GSE13601, GSE23036, and GSE27020) profiled on different versions of Affymetrix array chips and including a total of 358 tumor samples, were integrated following a meta-analysis approach through virtualArray R/BioConductor package [41]. These datasets were annotated and redundant probe sets were collapsed by EntrezID. Batch effects were removed using ComBat. The resulting integrated dataset was named MetaHNC-B.

### Unsupervised subtype discovery

Unsupervised tumor subtype identification on the MetaHNC-A was performed using k-means clustering of the most variant genes (*n* = 4950) and 1-Pearson correlation as distance matrix. In addition, the consensus unsupervised method as implemented in the R package was used in tumor subtype identification. ConsensusClusterPlus [42] has been applied to the data through 1000 re-sampling interactions by randomly selecting a fraction of the samples. We tested the existence of 2 < *k* < 10 clusters. In order to identify the number of clusters giving the maximum stability, empirical cumulative distribution function (CDF) plots displaying consensus distributions for each *k* was assessed. As stated in Monti [43], the choice of the number of clusters depends on the delta area plot and when the increase in the CDF area becomes equal to zero. To estimate the accuracy of the classification, Silhouette width values [44] for all the samples were calculated (R-package: cluster). Significance for each cluster was assessed in a pairwise fashion (R-package: SigClust) and reported as *p*-values [45]. An evaluation of sample size adequacy of the training set [46] was assessed according to Warnes and Liu (R-package: ssize) [47] and computed imposing type I error rate (FDR), *α* = 0.05 and minimum effect size (log fold-change), Δ = 1.

### Progression analysis of disease

In order to identify the relevant connections among the data, we applied Progression Analysis of Disease (PAD) [48], a tool able to unravel the topological characteristics of the data. This approach is an application of Mapper [18] that allows one to recognize local clusters within the data and assess the relationships among these small clusters. The output of the analysis collapses the data into a simple, low dimensional shape that summarizes the main features of the data.

### Statistical and bioinformatics methods

Statistical analysis was performed using R [49], version 2.15, BioConductor [50], release 2.10, and BrB-ArrayTool developed by Dr Richard Simon and the BRB-ArrayTools Development Team (v4.2.0; National Cancer Institute, USA).

Using Ingenuity Pathway Analysis (IPA 8.5, Ingenuity Systems, Qiagen, USA) and genes set enrichment analysis (GSEA) [51], we performed gene functional characterization. Through the IPA tool, the identified genes were associated with a canonical pathway in Ingenuity's Knowledge Base and used to analyze the signaling pathways, cellular location, function, and network connections. We performed GSEA with 2270 pathways including curated gene sets from pathway databases, publications on PubMed, genes based on Gene Ontology annotation and 179 oncogenic signatures present on Molecular Signatures Database (Broad Institute, USA).

PAM (Prediction Analysis for Microarrays) [52] was applied to identify a classifier in order to project our classification to other datasets. The prediction rule was computed on a selection of 40 core samples (the 40 cases for each subtype with greater positive Silhouette values). The classifier was applied to TCGA, GSE39368 and MetaHNC-B.

Since GSE686 and GSE39368 report the identification of potential molecular subtypes, these datasets were used to investigate the correspondence between their and our classification. To assess the degree of molecular correspondence Subclass Mapping (SubMap version 3, GenePattern Software; Broad Institute [53]) was applied on the genes entering into the PAM-classifier. This algorithm calculates the gene expression enrichment in each subtype between the training and the validations dataset providing a *p*-value indicating the significance of underlying molecular profiles.

Survival was analyzed in the GSE39368, MetaHNC-B, and TCGA datasets according to the Kaplan-Meier method and specific endpoints reported in each study (relapse free for GSE39368 and overall survival for TCGA). Differences between the six subtypes were assessed using log-rank test and R package survival.

The potential association of subtypes to four gene-expression signatures: radio-sensitivity index (RSI) [19]; 15-gene hypoxia classifier [20]; 13-gene signature for HPV-negative oral squamous cell carcinoma (OSCC) [21]; 172-gene model [22], was investigated. The genes were mapped using EntrezID annotation and, applying the algorithms described in De Cecco *et al*. [22], a value for each case in the TCGA and GSE39368 datasets was assessed and compared with the assigned six subtypes.

The association between clinical parameters and subtype membership was assessed through the ctree function present in party R package [54] using default parameters. The analysis was performed on TCGA and GSE39368 datasets.

Drug sensitivity was assessed through pRRophetic R package [55], following the pipeline established by the authors. This tool incorporates the public data from the Cancer Genomic Project [23] including baseline gene expression data and drug sensitivity on 700 cell lines. The analysis was carried out selecting ‘upper aerodigestive’ as tissue type. Microarray probes were mapped to the official GeneSymbol, cell line and MetaHNC-A datasets were homogenized using ComBat function and 20% of genes with lowest variability were removed. A linear ridge regression model was fitted to the homogenized dataset, yielding a drug sensitivity estimate for each tumor. ROC curves were estimated by pROC R package [56].

## SUPPLEMENTARY TABLES AND FIGURES




