# Supplementary material for: Head and neck cancer subtypes with biological and clinical relevance: Meta-analysis of gene-expression data
Source: Oncotarget. 2015 Mar 20;6(11):9627–42. doi: 10.18632/oncotarget.3301 (PMC4496244; doi:10.18632/oncotarget.3301)
Supplement: Supplementary file 1 [file oncotarget-06-9627-s001.pdf]

SUPPLEMENTARY TABLES AND FIGURES

Supplementary Table S1. Datasets used in the present study. The type of analysis was gene expression microarray in all studies, but the TCGA was performed by next generation sequencing.

Supplementary Table S2. SigClust analysis. *p*-values computed by SigClust R package for all pair wise comparisons among the six subtypes were reported

Supplementary Table S3. Composition of PAM classifier and predictive algorithm

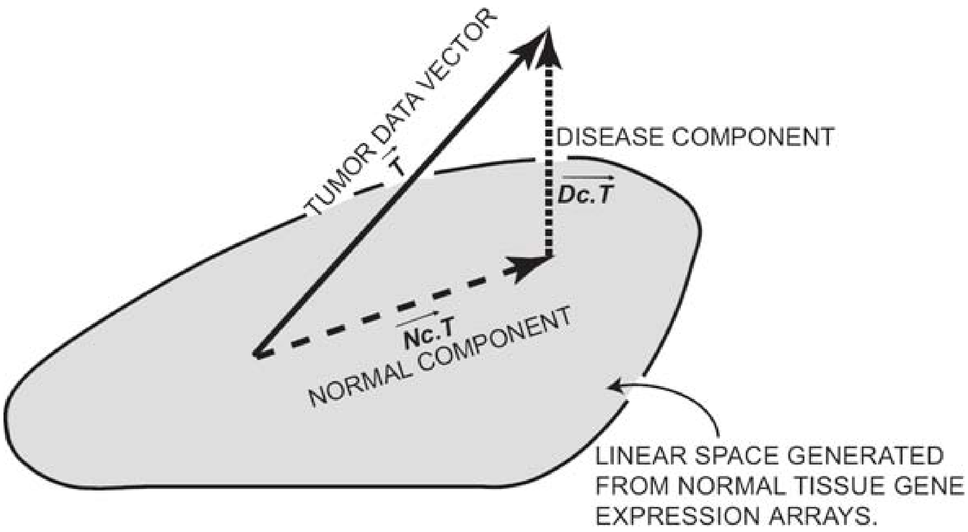

Supplementary Figure S1: Representation of DSGA. The cartoon illustrates the decomposition of tumor data vector into the normal and disease components. Copyright acknowledgement to "Monica Nicolau et al. Disease-specific genomic analysis: identifying the signature of pathologic biology Bioinformatics (2007) 23 (8): 957–965 doi:10.1093/bioinformatics/btm033, Fig. 1". Published by Oxford University Press. The figure is distributed under the CC BY-NC license.

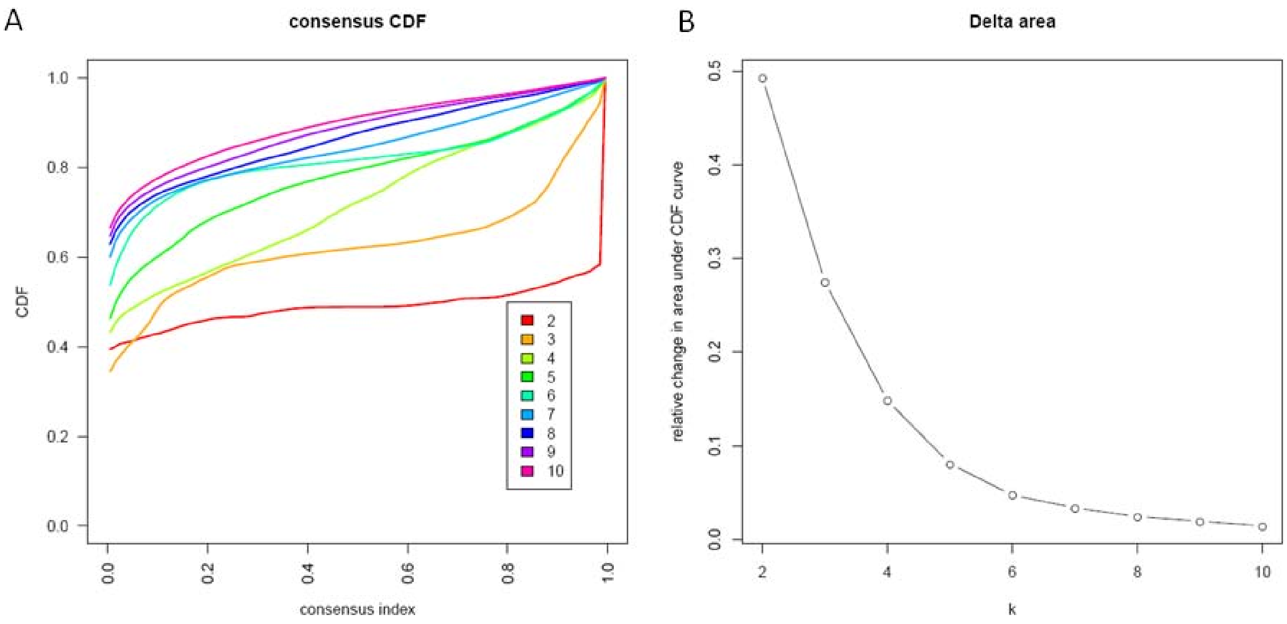

Supplementary Figure S2: Stability of the subtypes identified by ConsensusClusterPlus. A. Plot of consensus cumulative distribution functions (CDFs) for different numbers of clusters, ranging from 2 to 10. Large differences in the range $2 < k < 6$ shows greater stability associated to increasing numbers of clusters. An increase of k beyond 6 produces small gains. **B.** The plot shows the relative change in the area under the CDF curve.

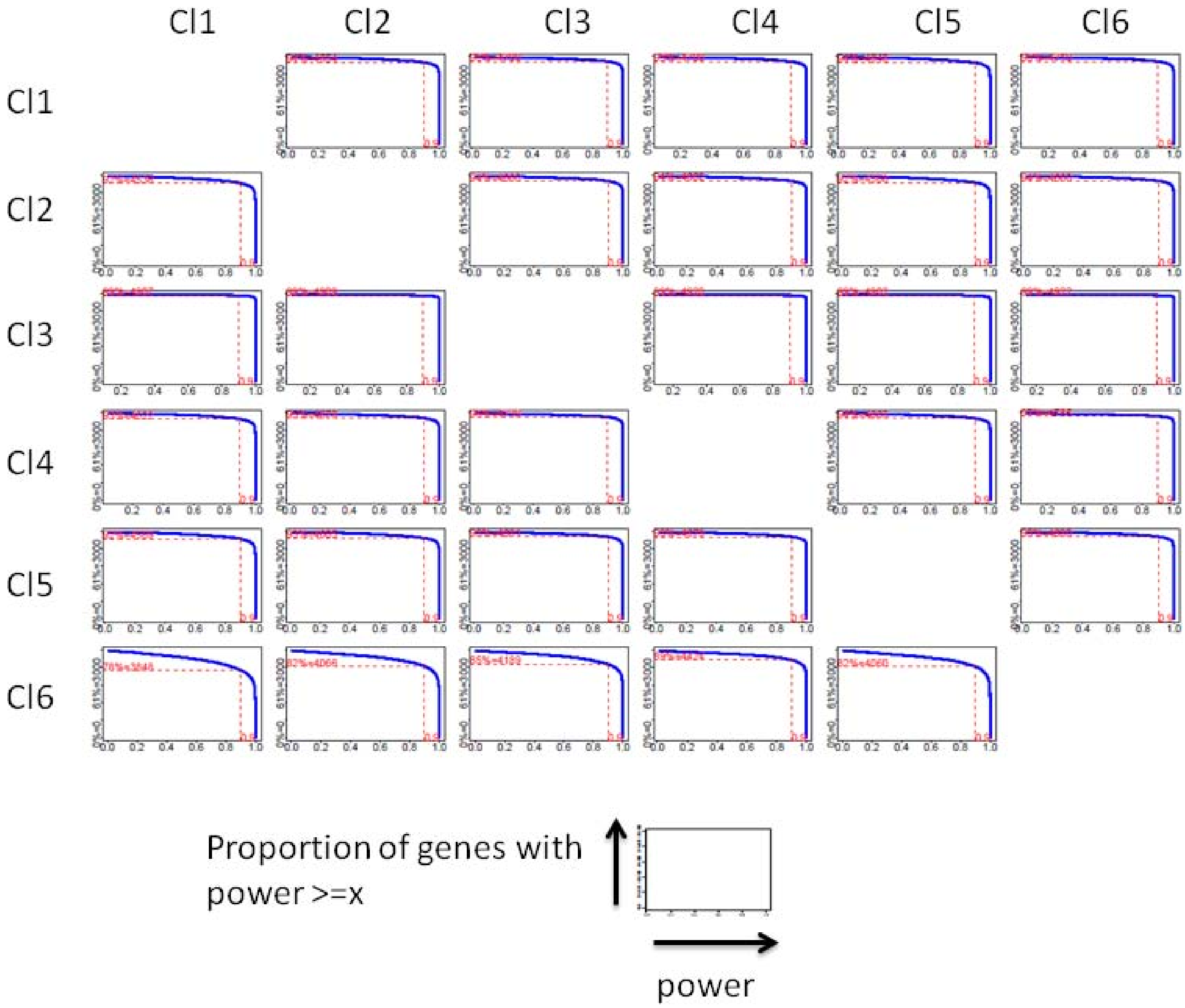

Supplementary Figure S3: Estimate sample size adequacy in MetaHNC-A. The plot displays the relationship between power for the genes present into MetaHNC-A and sample size defined by ConsensusClusterPlus (Cl1 = 89; Cl2 = 77; Cl3 = 154; Cl4 = 79; Cl5 = 81; Cl6 = 47). The percentage of genes achieving at least power = 90% is visualized following a pair-wise testing. The sample size of Cl1, Cl2, Cl3, Cl4 and Cl5 ensures that > 90% of genes reach 90% power. The sample size of Cl6 ensures that at least 78% of genes reach 90% power.

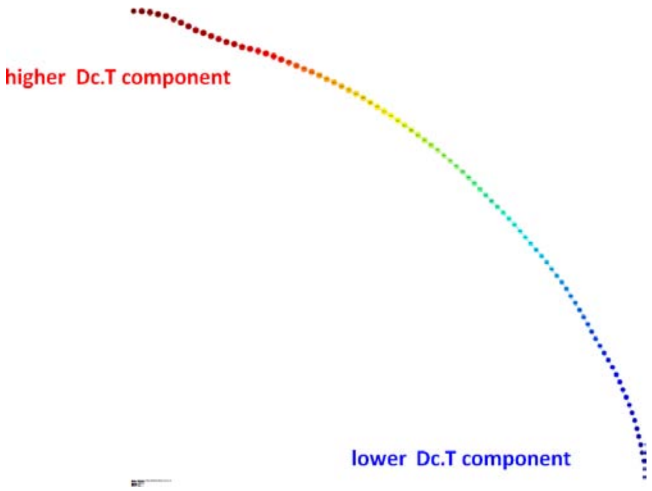

Supplementary Figure S4: Progression analysis of disease (PAD) applied to the HNSCC meta-analysis dataset. Each bin in the graph is a local cluster including a certain number of cases and they are ordered by the degree of variation from the normal state (HSM). Blue bins represent cluster of tumors showing the smallest deviation from HSM, while red bins are tumors whose deviation from HSM is the largest.

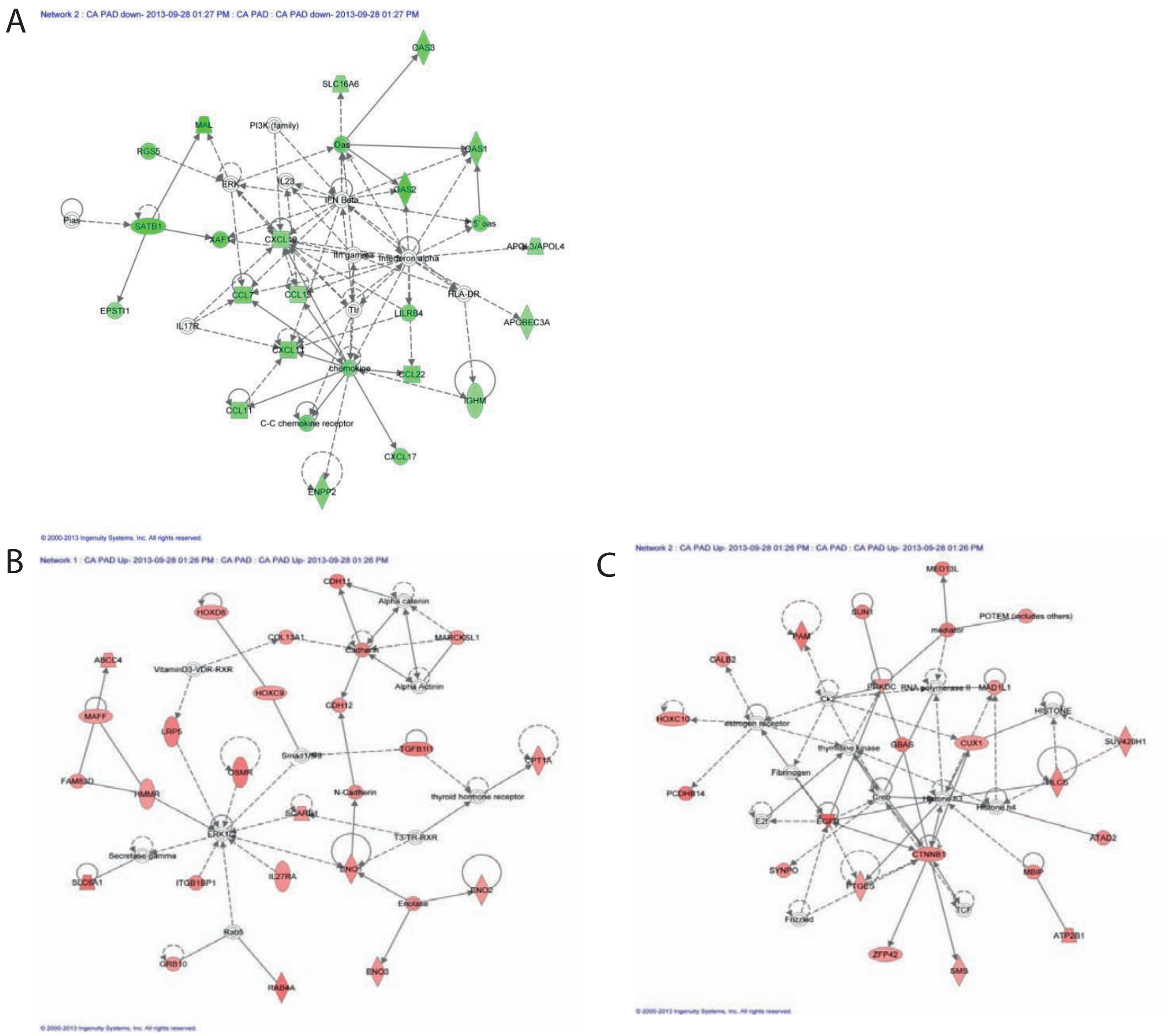

Supplementary Figure S5: Ingenuity Pathway Analysis (IPA) of genes correlated to PAD. The genes associated to PAD were divided in negatively and positively correlated and separately characterized by IPA. Imposing a significant score > 30 three networks containing negatively (panels A) and positively (panels B and C) correlated genes were identified.

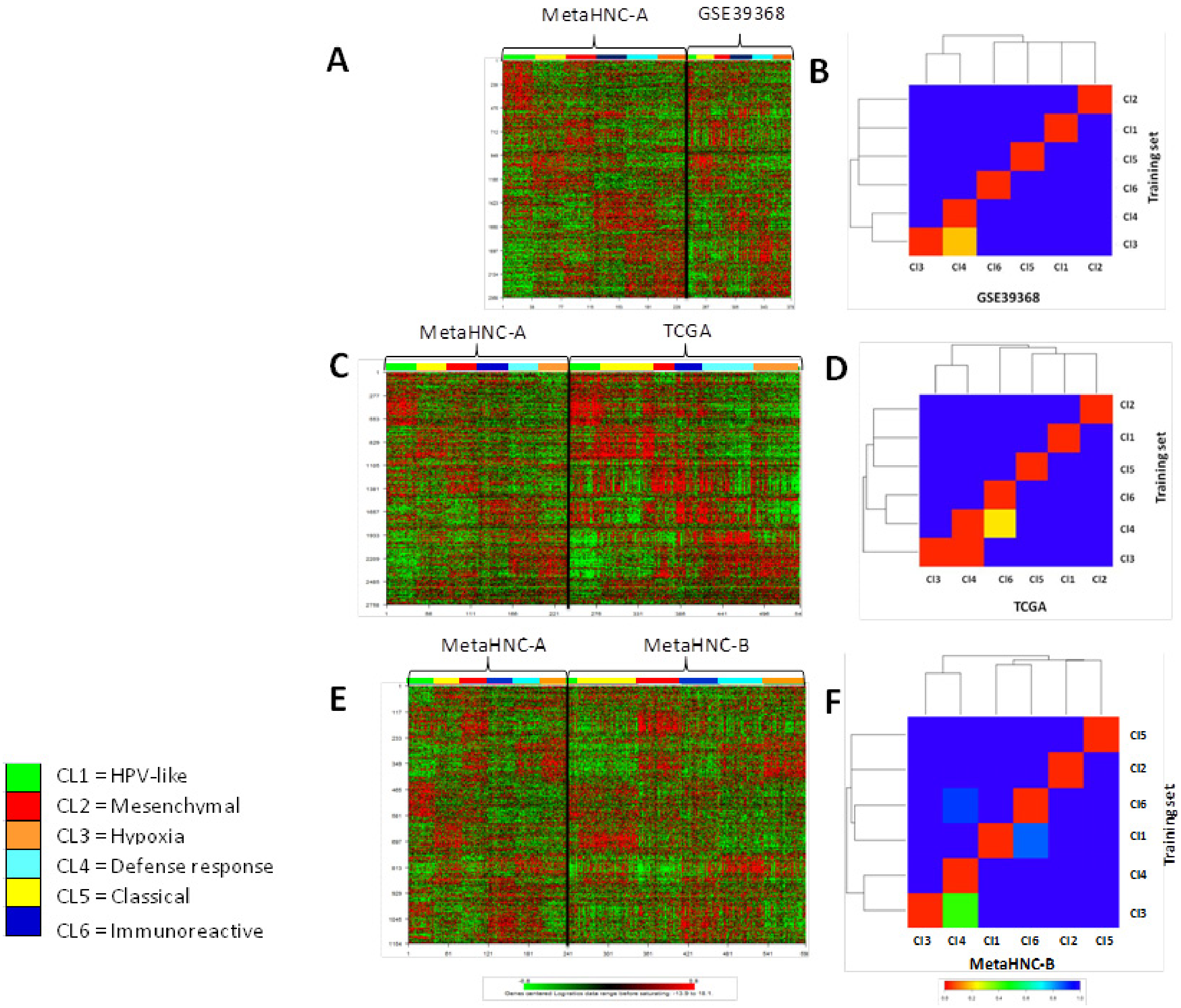

Supplementary Figure S6: Validation of the six subtype classification in two independent datasets. In the heatmaps, the order of classifier genes was maintained in both training and validation sets to show the expression similarities between datasets; the samples are ordered based on their predicted membership. The subclass mapping quantifies the significance of the similarity in expression patterns between training and validation sets. Red, high confidence for correspondence; blue, lack of correspondence. Heatmap and Submap on GSE39368 is shown in **A.** and **B.** respectively; heatmap and submap on TCGA is shown in **C.** and **D.** respectively; heatmap and submap on MetaHNC-B is shown in **E.** and **F.** respectively.

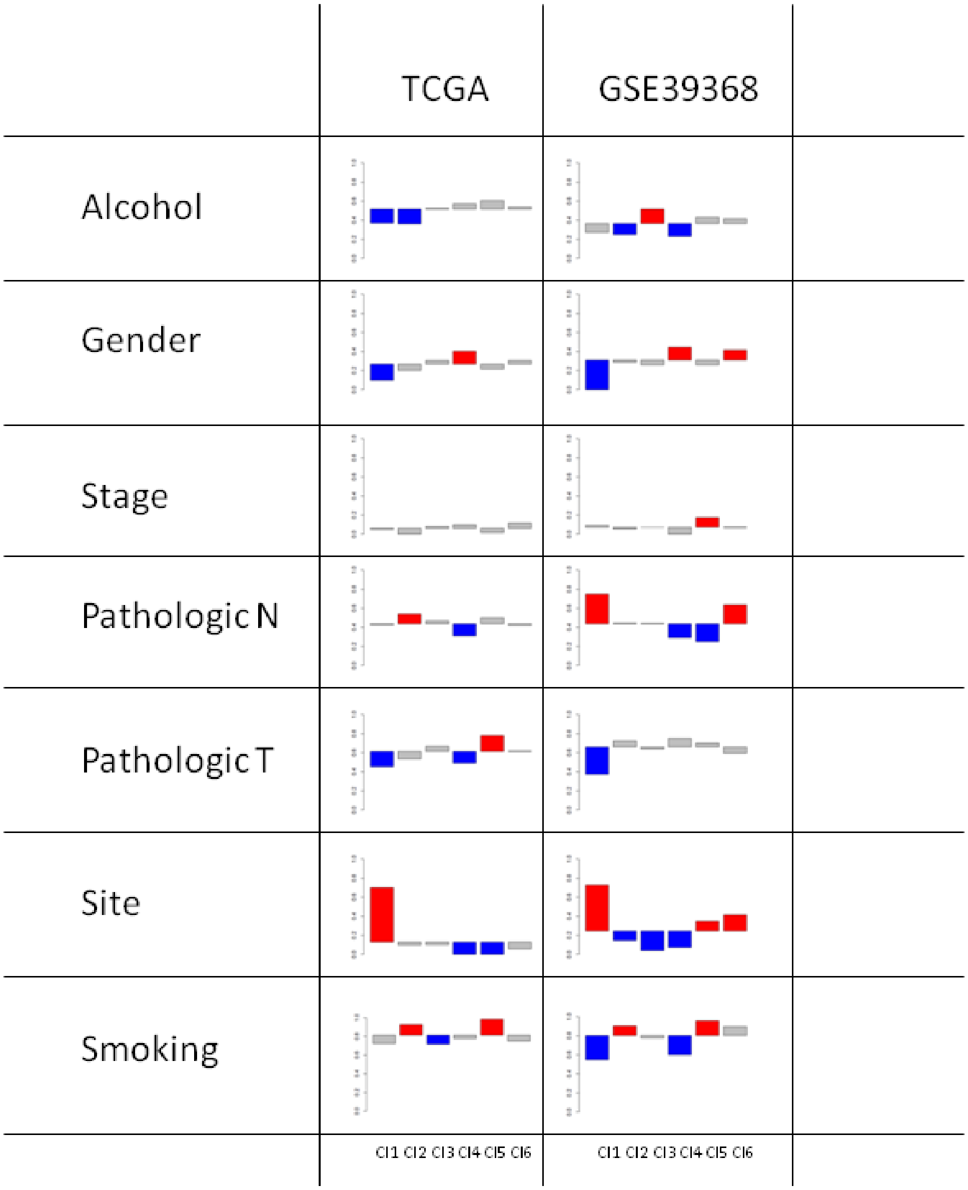

Supplementary Figure S7: Clinical characteristics of individual subtypes in the TCGA and GSE39368 datasets. Columns represent the subtypes and rows the clinical variables. The baseline is the average proportion of a variable in each dataset. In every subtype, red bars indicate an enrichment (> 10%), while blue indicate a depletion (< 10%) compared to the baseline. The variables are dichotomized as follows: alcohol = Heavy/none or light consume; gender = female/male; stage = I + II/III + IV; pathologic N = N2 + N3/N0 + N1; pathologic T = T3 + T4/T1 + T2; site = oropharynx/other sites; smoking = yes/no.

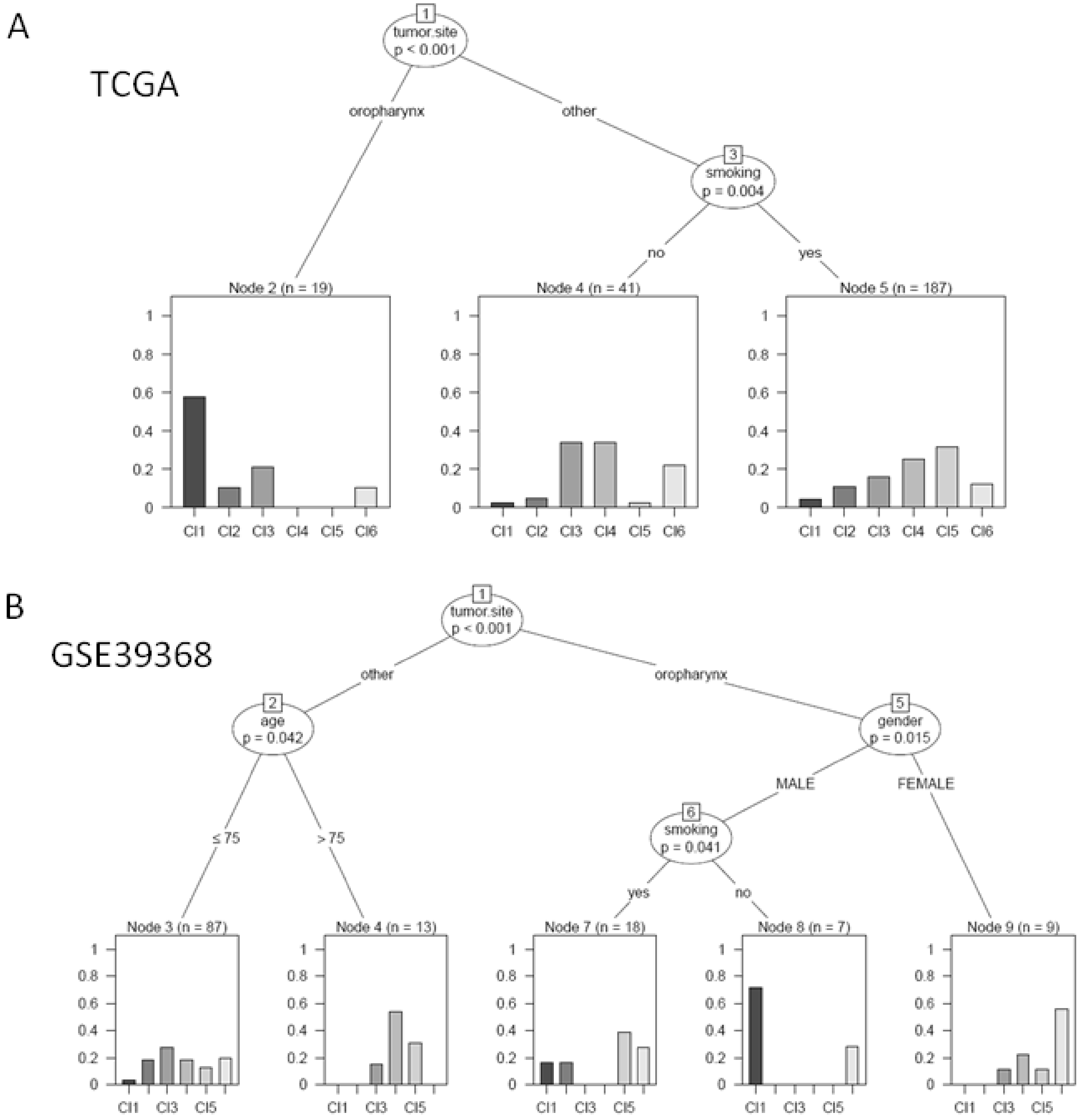

Supplementary Figure S8: Conditional inference tree based on clinical/pathological parameters. Five variables (i.e. gender, age, smoking history, pathologic stage, and site of primary tumor) were used to train a classification tree on datasets with complete data information available: **A.** TCGA, 247 cases; **B.** GSE39368, 134 cases.

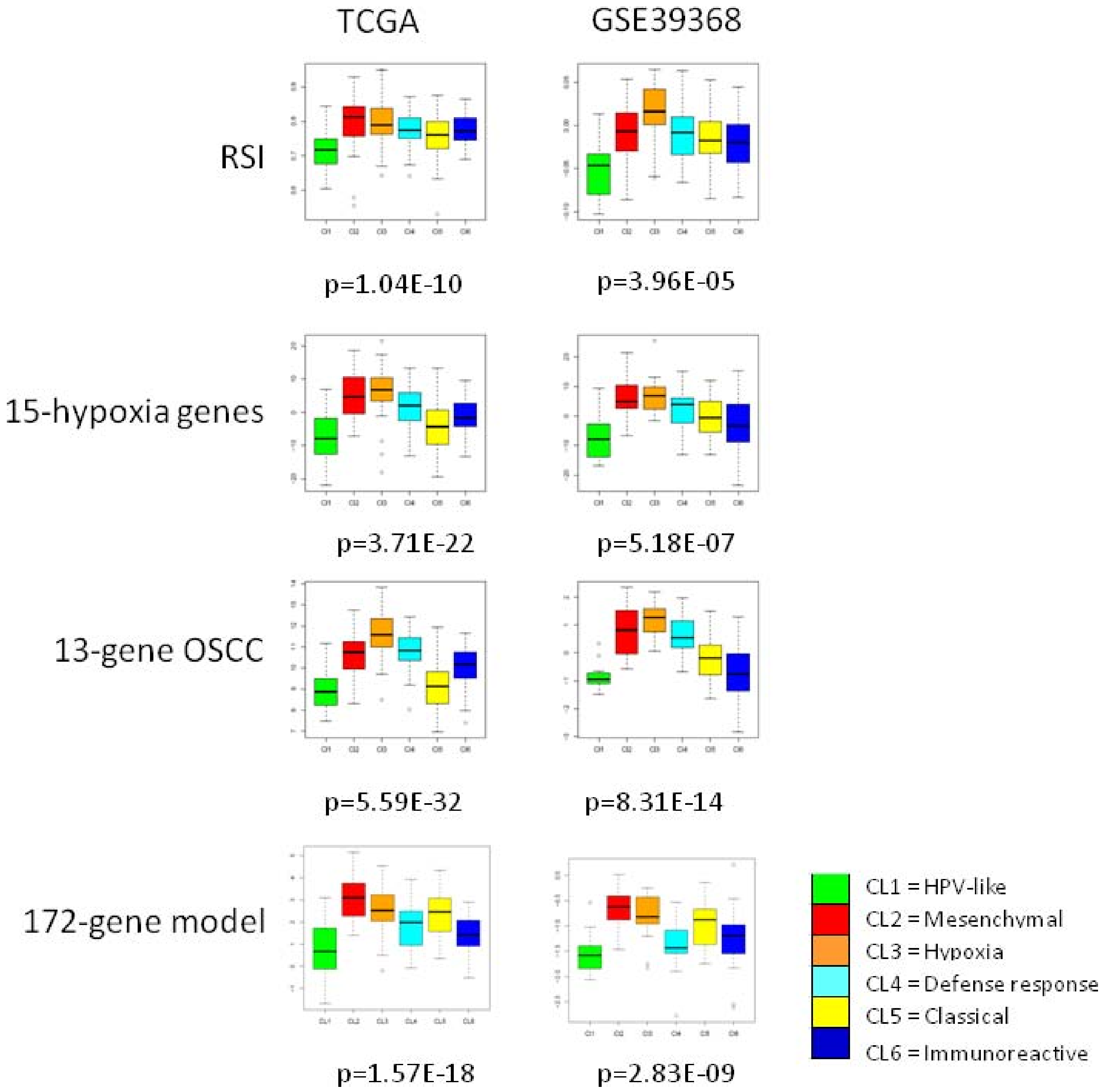

Supplementary Figure S9: Prognostic signatures and association to the six subtypes. Four published signatures were investigated for their relationship to the six subtype stratification on TCGA and GSE39368 datasets: (i) RSI; (ii) 15-gene hypoxia classifier; (iii) 13 gene OSCC HPV-negative signatures; (iv) 172-gene model. *p* = Kruskal-Wallis test.
